# The health, social and educational needs of children who have survived meningitis and septicaemia: the parents’ perspective

**DOI:** 10.1186/1471-2458-13-954

**Published:** 2013-10-10

**Authors:** Laura J Clark, Linda Glennie, Suzanne Audrey, Matthew Hickman, Caroline L Trotter

**Affiliations:** 1 School of Social and Community Medicine, University of Bristol, Bristol, UK; 2 Meningitis Research Foundation, Thornbury, Bristol, UK; 3 Now at: Disease Dynamics Unit, Department of Veterinary Medicine, University of Cambridge, Madingley Road, Cambridge CB3 0ES, UK

**Keywords:** Meningitis, Septicaemia, Sequelae, Aftercare, Survey, Qualitative

## Abstract

**Background:**

Survivors of bacterial meningitis and septicaemia can experience a range of after-effects. There is little published research on the needs and provision of aftercare for children surviving bacterial meningitis and septicaemia.

**Methods:**

Mixed methods study employing a survey and follow-up interviews with a sample of survey participants recruited from Meningitis Research Foundation’s member database and social media.

**Results:**

Of 194 eligible survey respondents, 77% reported at least moderate short-term after-effects, and 57% a need for aftercare or support. Most parents reported that their child received a hearing test (98%) and follow-up appointment with a paediatrician (66%). Psychosocial after-effects were most common and the greatest need was for educational support. About half of participants felt their children’s needs for aftercare were met. We conducted interviews with 18 parents. Findings suggest access could be limited by: parents’ inability to navigate systems in place, child’s age, and delayed identification of sequelae. Parents felt a comprehensive explanation of possible after-effects on discharge from hospital was required, and found uncertain prognoses difficult. Good communication between professionals enabled a service tailored to the child’s needs.

**Conclusions:**

Our study supports the NICE and SIGN guidelines and highlights areas for improvement in the aftercare of these children.

## Background

Bacterial meningitis and septicaemia remain an important cause of morbidity and mortality in the UK, despite progress in prevention through national immunisation campaigns against *Haemophilus influenzae* type b (Hib), meningococcus C and certain pneumococcal serotypes. Around 10% of cases will not survive and many survivors will be left with after-effects. A recent global review of the risk of disability after bacterial meningitis suggested that nearly 10% of survivors in Europe experienced major sequelae, including cognitive deficit, bilateral hearing loss, motor deficit, seizures, visual impairment and hydrocephalus [1]. In addition, psychological and emotional problems are common [2,3]. In cases where sepsis occurs, tissue necrosis may result in scarring, growth plate damage and amputations of digits and limbs [4,5] and permanent renal failure [6]. Septic shock is also linked to a greater likelihood of psychiatric disorder [7]. There are differences in the type and severity of sequelae according to the causative organism [1], with the highest prevalence of sequelae following pneumococcal meningitis [8]. There are even differences between strains of the same organism; particular meningococcal strains have been associated with poorer outcomes in the UK [9] and France [10].

Current UK guidelines on the management of bacterial meningitis [11] recommend a hearing assessment within four weeks of being fit to test for all children who have had bacterial meningitis or meningococcal disease, irrespective of apparent need, a further follow-up appointment with the paediatrician 4–6 weeks post-discharge; and that discharging clinicians should consider the need for aftercare for a range of neurological and physical sequelae. There is, however, little published information on the needs for aftercare of children surviving meningitis. This information is vital for providing adequate services for survivors. Information on aftercare needs and provision is also essential to allow the full costs of the disease to be accounted for in economic analyses of the impact of vaccination and other preventive measures.

We designed a multiple choice questionnaire to ask parents of children who survived meningitis or septicaemia about the aftercare services that were required and whether they could access those services. We also employed qualitative research methods to enhance the survey data, using one-to-one interviews to explore parents’ views about aftercare services. The mixed methods approach of this study allowed us to gain a more comprehensive understanding of parents’ and children’s needs and experiences when accessing follow-up services.

## Methods

### Study design

Eligible participants were the parent/legal guardian of children (aged <18 years at the time of illness) who had survived meningitis or septicaemia between January 2000 and May 2010, living in the UK or Ireland. Members of Meningitis Research Foundation (MRF), individuals with experience of meningitis and septicaemia, were sent a targeted email invitation or letter and a participant information sheet. We did not contact members whose experience was within six months to prevent causing further distress. A more general invitation was placed in MRF’s e-newsletter and social media websites. Participants could complete an online survey or request a paper format of the questionnaire. 334 questionnaires were completed. We excluded participants who did not come from the UK or Ireland (N = 21), were not the parent or legal guardian (N = 89), had experienced disease prior to 2000 (N = 14) or had experience of adult illness (18 years old or more at the time of disease; N = 16). After exclusion of non-eligible participants the survey sample consisted of 194 parents. Ethical approval was gained from the Research Ethics Committee of the University of Bristol Faculty of Medicine and Dentistry, and all participants provided informed consent.

Stage one, the questionnaire (available on request from the corresponding author) was designed to elucidate disease history, which services were required by children after meningitis and septicaemia, whether follow-up was offered according to the National Institute for Health and Clinical Excellence (NICE) guidelines [11], how easy it was to access services, and parental opinion of the care provided in terms of usefulness and satisfaction. The language and multiple choice questions were informed by a previous member survey, consultation with specialists in the different areas of care, and a piloting process involving 10 MRF members. The terms ‘useful’ and ‘happy’ were used in two questions at the end of each section of the questionnaire in an attempt to understand how well the child’s needs were met. These terms were deemed most appropriate in response to the participants comments when we piloted the questionnaire. Many parents said that the treatment they received did not necessarily improve the situation for their child (i.e. usefulness) but that they were still happy with the care they received.

In stage two, the follow-up interviews, a sample of participants who had consented to be interviewed were contacted. Sampling was purposive and only those parents reporting permanent after-effects, and who had accessed aftercare and support, were invited for interview, based on their answer to the survey question, ‘Overall, to what extent do you feel the aftercare and support has met/meets your child’s needs?’ We selected parents of children with a range of causative organisms, allowing us to explore aftercare for neurological and physical sequelae. Eighteen parents were interviewed, either face-to-face in their homes (n = 9) or by telephone (n = 9). The interviews were semi-structured, beginning with an open question inviting parents to provide a narrative background of their child’s illness leading up to them requiring aftercare. Further questions explored parents’ opinions of the care their children received. All but one of the interviews were digitally recorded and fully transcribed. The transcripts and researchers notes were anonymised.

### Analysis

Descriptive statistics were primarily used in analysis of survey data. Multivariable logistic regression was used to examine associations between permanent sequelae and causative organism (specifically pneumococcal disease compared with other organisms). We used Stata v.10.1 for all quantitative analysis.

Qualitative analysis employed the constant comparison method from grounded theory [12-14]. Transcripts were read individually and units of text were coded using terms relevant to the participants’ experiences and the research question. The coded transcripts were scrutinised for differences and similarities within emerging themes, keeping in mind the context in which these arose.

## Results

### Survey results

#### Participant characteristics

There were 194 eligible survey respondents. The mean age of children at the time of illness was 3 years 10 months and median time since illness was 5 years. The majority of respondents were from England (75%) with the remainder from other parts of the UK (22%) and Ireland (3%).

#### Sequelae

Most parents reported that their child had at least moderate short term after-effects with 23.2% reporting no after-effects at all (Table 1). Most frequently reported problems were behavioural, psychological or emotional, referred to henceforth as psychosocial sequelae (40.7%, Table 1). Permanent effects were more likely to occur in children with pneumococcal disease than with any other disease type, after adjusting for age when ill and disease form (OR 5.1, *P* = 0.01, 95% CI 1.5-16.8).

**Table 1:** Characteristics of survey participants, including illness, sequelae and aftercare requirements

	**n**	**%**
**Disease form**		
Meningitis	76	39.2
Septicaemia	16	8.3
Both meningitis and septicaemia	102	52.6
Total	194	
**Causative agent**		
Meningococcal	68	35.1
Pneumococcal	49	25.3
Other bacterial	30	15.5
Viral	4	2.1
Fungal	6	3.1
Unknown	37	19.1
Total	194	
**Severity of after-effects**^**1**^		
No after-effects	45	23.2
Moderate short term	14	7.2
Severe short term	31	16.0
Moderate permanent	43	22.2
Severe permanent	39	20.1
Moderate and severe permanent	1	0.5
Too soon to tell if permanent	6	3.1
Too soon to tell if any	15	7.7
Total	194	
**Sequelae**^**2**^		
Behavioural, psychological or emotional	79	40.7
Fatigue (affecting day-to-day activities)	50	25.8
Recurrent severe headaches	33	17.0
Sensory (hearing loss/balance problems/visual loss or disturbance)	37	19.1
Motor or coordination problems	29	15.0
Epilepsy or other seizure disorder	17	8.8
Hydrocephalus	7	3.6
Brain damage	37	19.1
Scarring/tissue or muscle damage/long term wounds	31	16.0
Joint or limb pain/arthritis	35	18.0
Amputations or growth plate damage	14	7.2
Organ damage	7	3.6
**Aftercare or support service needed**^**3,4**^		
Educational support	59	30.4
Physiotherapy	49	25.3
Speech and language therapy	38	19.6
Behavioural, psychological or emotional support	36	18.6
Occupational therapy	30	15.5
Hearing follow-up	26	13.4
Neurology	26	13.4
Child development centre services	24	12.4
Plastics	12	6.2
Orthopaedics	12	6.2
Prosthetics	6	3.1
Treatment for hydrocephalus (CSF chunt)	5	2.6
*No aftercare or support needed*	83	42.8
*Only one aftercare or support service needed*	28	14.4
*>1 aftercare or support service*	83	42.8

^1^Parents could select more than one category under severity of after-effects, here we have only counted the most severe category a parent selected.

^2^Parents could report more than one type of sequelae per child so the total here exceeds 194, but the percentage is based on the proportion of the sample (x/194) reporting that after-effect.

^3^This includes parents who chose the answer ‘My child will need this in future’.

^4^Parent could report more than one type of aftercare per child so the total here exceeds 194, but the percentage is based on the proportion of the sample (x/194) reporting that aftercare need.

#### Requirement for and provision of aftercare

Only 2% of those with bacterial meningitis/meningococcal septicaemia were not offered a hearing assessment at all, and 51% were offered an assessment within 4 weeks of being fit to test (as recommended by NICE). Two thirds were offered a follow-up appointment with a paediatrician after coming home from hospital although 20.1% were not offered a follow-up appointment at all (of whom 31% (12/39) reported no after-effects).

Most parents reported that their child required aftercare and support (Table 1). The greatest need was for educational support (30.4%) and this proportion was higher (52.5%) amongst children with psychosocial sequelae. Most people could access the follow-up services they needed. For hearing (n = 25), speech and language therapy (n = 36), occupational therapy (n = 49), behavioural, psychological or emotional support (n = 31) and child development centre support (n = 23) around half of respondents (range 48% to 56% depending on service) had no difficulty accessing aftercare. This was higher for physiotherapy (n = 49) at 69% and for plastic surgery (n = 12) at 83%. However for every category of aftercare and support, excepting plastic surgery, at least 20% had some difficulty or could not access services at all. For those services required by fewer children there was more variation in how easy it was for parents to gain access. For those requiring plastic surgery (n = 12) there was either no difficulty or parents could not gain access at all. For parents whose child required prosthetics (n = 6) only one gained access with no difficulty. Of the 59 parents who reported the need for educational support, 5 (8%) did not receive any and 26 (44%) did not receive enough. Some parents reported having to pay for aftercare themselves because of difficulties in accessing state services, but this was fairly infrequent (1 to 3 cases per category).

#### Parents’ satisfaction with aftercare provided

About half of participants felt their children’s needs were being met, and half did not. Most parents found aftercare and support services useful, with the exceptions of psychosocial support, educational support and prosthetics (Table 2). There were no parents who reported that prosthetics (i.e. the equipment provided) were useful but 40% of them were happy with the support given by staff. Most agreed that they were happy with the care received (Table 2). Again parents were more likely to be unhappy with educational and psychosocial support, and prosthetics follow-up. In most cases, if parents felt services were useful they were also happy with the care provided.

**Table 2:** Percentage responses to statements a) ‘The aftercare/support my child received has been useful or successful’ and b) ‘I am happy with the aftercare/support my child my received’

**a) Aftercare useful/successful**	**H**	**S**	**PH**	**OT**	**PL**	**O**	**PR**	**B**	**E**	**N**	**C**
Strongly disagree	12.5	13.5	4.8	4.2	8.3	0	50.0	7.4	17.5	7.7	10.0
Disagree	0	10.8	7.1	4.2	8.3	0	16.7	22.2	19.3	3.9	10.0
Neither agree nor disagree	12.5	16.2	14.3	16.7	16.7	16.7	33.3	33.3	15.8	19.2	25.0
Agree	50.0	40.5	33.3	54.2	16.7	41.7	0	25.9	31.6	57.7	45.0
Strongly agree	25.0	18.9	40.5	20.8	50.0	41.7	0	11.1	15.8	11.5	10.0
Total responded to question (N)	24	37	42	24	12	12	6	27	57	26	20
**b) Happy with aftercare/support**	**H**	**S**	**PH**	**OT**	**PL**	**O**	**PR**	**B**	**E**	**N**	**C**
Strongly disagree	16.7	19.4	2.6	0	9.1	0	60.0	6.9	18.5	7.7	10.5
Disagree	8.3	13.9	12.8	18.2	18.2	9.1	0	34.5	16.7	11.5	10.5
Neither agree nor disagree	8.3	13.9	12.8	9.1	9.1	18.2	0	20.7	18.5	11.5	26.3
Agree	41.7	33.3	25.6	40.9	9.1	27.3	20.0	20.7	29.6	53.9	42.1
Strongly agree	25.0	19.4	46.2	31.8	54.6	45.5	20.0	17.2	16.7	15.4	10.5
Total responded to question (N)	24	36	39	22	11	11	5	29	54	26	19

**Key: H** = hearing follow-up, **S** = speech and language therapy, **PH** = physiotherapy, **OT** = occupational therapy, **PL** = plastic surgery, **O** = orthopaedics, **PR** = prosthetics, **B** = behavioural psychological or emotional support, **E** = educational support, **N** = neurology or epilepsy team, **C** = child development centre.

### Interview findings

To illuminate some of the findings from the survey, and further explore some of the issues raised, follow-up interviews were conducted. The 18 interview participants (P1-P18) were from a range of socioeconomic classes and professions. Low numbers meeting inclusion criteria and difficulty in arranging interviews meant that participants were from England, Scotland and Wales only. Their affected children were at various points in follow-up after meningitis or septicaemia, with a range of physical, cognitive and emotional needs. Services accessed included physiotherapy, occupational therapy, educational support, orthopaedics, neurology, visual impairment services, audiology, and speech and language therapy. We identified two main themes: accessing appropriate support and follow-up care and communication.

### Accessing appropriate support and follow-up care

#### Navigating the system

Most parents could access the aftercare or support service their child needed, although sometimes with difficulty. Only one parent said they could not gain access to a service at all. Learning to navigate the support systems in place was a common issue that emerged. Many parents felt that they had to ‘learn the language’ and when coming home from hospital parents did not know ‘what to do next’. This was also the case when applying for disability living allowance and accessing respite social care.

There was a sense that parents felt they had to do things themselves and sometimes it was easier to do it that way, both in terms of finding out how to access support and in gaining provision. For parents who did not find it difficult to navigate the systems in place, organisational barriers had been overcome. Often there was a key point of contact who was ‘proactive’ and instigated further appointments.

Almost all parents interviewed had experienced difficulties in gaining sufficient or timely care. This was reported to be due to a lack of staff or restricted budgets. In cases where the child had a statement of educational needs the school could prove extremely useful in provision of services, making access to aftercare and support more frequent, with less delay and over a long enough period of time.

P1: “Because her needs are so complicated and they’re in so many different areas… there is physio, speech and language, OT, neurology…so many different people for us to learn, to keep up with and to learn the language, we didn’t know what to ask…we’re just completely … overwhelmed.”

P4: “She was told she would only have thirty five per cent hearing, but then told that she couldn’t at that time apply for a hearing aid because she was borderline… so we went ahead and got one for her.”

P8: “He’s now gone into a specialist educational provision and now because they’re on-site he’s kind of accessing all those services again on a really regular basis.”

#### Young age as a barrier to gaining a clear diagnosis and support

Gaining access to services was often difficult when the child was very young. This could be because of difficulty testing young children (as in the case of hearing assessments), or because disabled children may be perceived to have similar needs to very young children. Regular check-up appointments were often mentioned in examples where young age did not present a barrier to diagnosis or access.

P1: “[Social worker] wrote to say that [her] needs were no greater than a child of her own age…it was very clear to see when she came to visit us that, she can’t move, she can’t talk, we have equipment all over our house…that was a huge thing to us, they said ‘yes she meets our criteria but that doesn’t mean she meets the criteria for services’.”

#### Poorly appreciated link between meningitis and sequelae

The less visible, psychosocial and cognitive after-effects of meningitis often made it hard to access support at school and there was little appreciation of the link between meningitis and long term psychosocial after-effects. Parents felt that the link between acute meningitis and long term complications was poorly understood and addressed by the health and social care system. This consequential inability to categorise children made accessing services harder.

P2: “You look at him against all his other class and you wouldn’t straight away say this is the child who’s had meningitis, this is the child who can’t hear in one ear, this is the child who struggles in these areas of social behaviour …so just trying to access any extra help in school is like pulling teeth.”

#### Appropriateness of support and aftercare

Where parents were unhappy with the support and aftercare offered, this was often because it was perceived as not fit for purpose: for example, prosthetic limbs were found to be too heavy for children to use, or the support offered was not tailored with the needs of the child in mind. Appropriateness of services depended on how much time and attention parents felt was paid to their child’s individual needs. Some parents felt that this was adequate while others did not.

P1: “… she has a helmet from orthopaedics because of her epilepsy…it fits poorly and she pushed it back so the bit of the head it’s supposed to protect, it doesn’t protect. I went back and said, ‘is there something better we can do with it?’ , and she said, ‘no that’s it’. Really, she cannot be the only child to be doing this.”

P2: “They spent a lot of time on his spatial awareness, and those types of things because he does seem to be quite clumsy…they picked up this constant need he has of stimulation to the head, which I hadn’t noticed.”

### Communication

#### Debrief before discharge

It may be difficult for health professionals to predict the likelihood of cognitive after-effects at the time of discharge, particularly in young children who are still to reach key developmental milestones. This often posed a real challenge to parents and was a source of worry and distress. Often parents were not ‘warned’ or told that there could be potential cognitive and behavioural after-effects, others were told to ‘wait and see’. Parents felt a lot of the frustration and distress may have been reduced if there had been better, more standardised ways of communication.

P3: “[Hospital] said, ‘he might be ok you know he might have problems, but you won’t know at the moment’…which I felt wasn’t really helpful either because it was kind of like well you have to go home and you just wait and see how he turns out…I don’t think I had the right support for that.”

P12: “I don’t know if there [is] something standard on discharge that parents are given, a booklet or something like that would have been so useful…I didn’t know of any time scales or what things I should be looking for.”

#### Involving parents

Parents wanted to be involved and informed about their child’s care and support, and often worried about their child being able to reach their potential. The expectations of the child differed between parents, school teachers or health professionals and there seemed to be little management of this aspect of aftercare. In cases where the parents felt listened to and involved, the care package appeared more tailored to the needs of parent and child.

P3: “I asked for certain things and he [said], ‘well he’s doing fine, it will be fine, it will be fine, and I think their expectations are too low to be honest’.”

P7: “The fact that he’d had an assessment [at school] and I don’t know what the outcome is… I don’t know if that’s in anyway had any bearing on what’s happening with him now.”

P13: “Yeah I think they’ve listened to whatever we thought about, you know we’ve always been of the mind that we wanted [him] to be as independent as he can be and so they’ve worked with that.”

#### Communication between professionals

Poor communication between different specialists resulted in support that was unresponsive to the child’s needs. When professionals did communicate, parents felt that there were shared plans and goals which facilitated meeting their child’s needs. Multidisciplinary team meetings involving parents, school staff and health visitors enhanced communication and cooperation in meeting the needs of the child.

P15: “They’ve just given her some words to practise, she doesn’t say the endings of any of the words …probably because she can’t hear them…speech and language can’t sort her hearing out, they can just try and help her with pronouncing the words, but if she can’t hear them then they’re hitting their heads against a brick wall.”

Interviewer: “Do speech and language and the audiology people, do they talk to each other?”

P15: “No, no.”

P13: “… and nothing was ever planned without [consultant]’s say so…to me that said we have got your son’s best interests at heart we have a plan and we know what we’re doing.”

## Discussion

Most parents who completed the survey reported that their child had either moderate or severe permanent after-effects, most common of which were psychosocial problems. Educational support was the most common support service required, but nearly half (44%) of those requiring this support did not get enough, and 8% did not receive any support. Most parents could eventually access all aftercare services, albeit with some difficulty or delay, but there was more variability in ease of access for plastic surgery and prosthetics which were needed by the fewest children. Parents felt that educational support, support for psychosocial problems, and prosthetics follow-up were least useful and these were also the services parents were least happy with in terms of the care provided. Overall around half of survey respondents reported that their child’s needs were met, and half stated that their child’s needs were not fully met.

The interviews provided richer data and possible explanations as to why parents were not happy with follow-up, or why services were not useful. Two main themes emerged:, accessing appropriate support and aftercare and communication. Access could be limited by a parent’s ability to navigate the systems in place, the level of service provision, the age of the child delaying identification of sequelae and related follow-up, and a poor appreciation of the link between meningitis and after-effects. Parents felt that a comprehensive debrief about the risks and range of possible after-effects on discharge from hospital was required, and found uncertain prognoses difficult to deal with. Good communication between professionals underpinned care and support that was responsive, tailored and took into account all of the child’s needs.

Many of the themes identified here are also reported from research on children surviving traumatic brain injury, including problems of access, communication, and navigating the systems [15-17]. The lack of educational support for children after meningitis evident here echoes the findings of de Louvois et al. [18] who reported inadequate educational support in meningitis survivors: only one-fifth of meningitis survivors who did not attempt any GCSEs had a statement of special educational needs.

Although basing this study on MRF members had a number of pragmatic advantages, our respondents had a much higher occurrence of after-effects than would be expected from a random sample of families affected. This may mean that our sample is not entirely representative of all survivors of bacterial meningitis. This focus did, however, enable us to efficiently examine families’ experiences of aftercare and its provision, which was our primary aim. It is possible that our respondents are not representative of all parents living with children surviving meningitis, and because they are involved with MRF may have different experiences of aftercare than other parents. To widen the sampling frame we used social media sites to publicise the survey, which is likely to have increased the number of respondents but made it difficult to estimate the response rate. Although the sample size for the qualitative interviews did not allow for complete data saturation, the themes identified here were recurrent. Further interviews may have provided more examples where needs were or were not met but we did not feel that this would substantially alter the findings presented here.

This research adds to the literature on the need for, and experience of, aftercare following meningitis and septicaemia. A recent study of parent’s experiences of support during and after their child’s diagnosis of meningococcal serogroup B disease [19] came to broadly the same conclusions as our own study, particularly the need for better communication throughout the treatment pathway. In an attempt to address these information and communication issues, Meningitis Research Foundation in conjunction with the Meningitis Trust have recently produced a booklet “Your guide: Recovering from childhood bacterial meningitis and septicaemia” and an accompanying patient journal to help families work together with health professionals to identify and deal with the physical and psychological after effects of meningitis and septicaemia as recommended by NICE. There are few other published papers on the needs of children surviving meningitis and septicaemia, in terms of aftercare and support services, perhaps due to the diversity of potential sequelae. The scope of this diversity is illustrated by the national guidelines for England and Wales [11] and Scotland [20] which recommend follow-up to assess children’s needs for referral for hearing loss, orthopaedic complications, skin complications, psychosocial problems, neurological and developmental problems and renal failure.

Although many children make a full recovery from meningitis and septicaemia, some experience considerable problems after recovering from the acute phase of the illness. Parent’s satisfaction with aftercare and support services was variable and our research highlights several areas for improvement. The experience of many of the families in this study predates the NICE and SIGN guidelines, which provide a clear framework for assessing aftercare needs. Our findings provide additional support for these recommendations. In particular, we identified a need for a comprehensive debriefing meeting with the discharging doctor to explain the potential long term after-effects to parents, and an explicit strategy for helping parents cope with an uncertain prognosis for their child. This may include frequent assessments for young children who present with ambiguous problems where a clear diagnosis is not always possible.

## Conclusions

Our study supports the NICE and SIGN guidelines for assessing the needs of children following meningitis and septicaemia and highlights areas for improvement in the aftercare of these children.

## Competing interests

Linda Glennie works for Meningitis Research Foundation, a charity that funds research, supports people affected and works to improve meningitis prevention, recognition, treatment, and aftercare through information and awareness campaigns. Laura Clark was jointly appointed by the University of Bristol and Meningitis Research Foundation.

## Authors’ contributions

LJC, LG, CT, MH conceived the study. LJC obtained ethical approval, set up and conducted the questionnaire and interviews with support from LG, CLT, MH. CLT supported the statistical analysis. SA supported the analysis and interpretation of the qualitative research. LJC wrote the first draft of the paper. All authors critically reviewed the findings and contributed to the writing of the manuscript. All authors read and approved the final manuscript.

## Pre-publication history

The pre-publication history for this paper can be accessed here:

http://www.biomedcentral.com/1471-2458/13/954/prepub
